# Moderate Immune-Related Liver Injury Is a Good Factor in Patients with Hepatoma Under Atezolizumab Plus Bevacizumab

**DOI:** 10.3390/cancers17193157

**Published:** 2025-09-28

**Authors:** Tai-Chi Wu, Po-Ting Lin, Wei Teng, Eric Yi-Liang Shen, Chung-Wei Su, Yi-Chung Hsieh, Wei-Ting Chen, Tsung-Han Wu, Chen-Chun Lin, Shi-Ming Lin, Chun-Yen Lin

**Affiliations:** 1 Department of Gastroenterology and Hepatology, Chang Gung Memorial Hospital, Linkou, Taoyuan 333, Taiwan; mp2347@cgmh.org.tw (T.-C.W.); linpoting01@cgmh.org.tw (P.-T.L.); mp1518@cgmh.org.tw (C.-W.S.); cutebuw@yahoo.com.tw (Y.-C.H.); weiting@cgmh.org.tw (W.-T.C.); lincc53@cgmh.org.tw (C.-C.L.); lsmpaicyto@cgmh.org.tw (S.-M.L.); 2 College of Medicine, Chang Gung University, Taoyuan 333, Taiwan; pts@cgmh.org.tw (E.Y.-L.S.); domani@cgmh.org.tw (T.-H.W.); 3 Liver Research Center, Chang Gung Memorial Hospital, Linkou, Taoyuan 333, Taiwan; 4 Department of Radiation Oncology and Proton Therapy Center, Chang Gung Memorial Hospital, Linkou, Taoyuan 333, Taiwan; 5 Clinical Metabolomics Core Laboratory, Chang Gung Memorial Hospital, Linkou, Taoyuan 333, Taiwan; 6 Department of Surgery, Chang Gung Memorial Hospital, Linkou, Taoyuan 333, Taiwan; 7 Department of Gastroenterology and Hepatology, New Taipei Municipal Tu Cheng Hospital (Built and operated by Chang Gung Medical Foundation), New Taipei City 236, Taiwan

**Keywords:** immune-related liver injury, ALBI grade, locoregional therapy, overall survival, progression-free survival

## Abstract

Background: Atezolizumab plus bevacizumab is the standard first-line therapy for unresectable hepatocellular carcinoma (uHCC). Immune-related liver injury (IrLI) is common; however, the association between IrLI severity and patient outcomes remains unknown. This study aimed to investigate the prognostic value of irLI in such patients. Methods: One hundred and sixteen patients who fulfilled the IMBrave150 inclusion criteria were enrolled. IrLI was defined as an increase in serum ALT and/or AST levels attributed to treatment and was graded according to the National Cancer Institute Common Terminology Criteria for Adverse Events v5.0. Results: A total of 61 patients (52.6%) developed any grade of irLI, with a median onset time of 1.7 months. Multivariate analysis revealed that grade II ALBI (hazard ratio [HR] = 2.003, *p* = 0.028) and BCLC stage C (HR = 3.876, *p* = 0.016) were associated with worse OS and PFS (HR = 1.327, *p* = 0.044 and HR = 1.790, *p* = 0.039, respectively), whereas grade 2 irLI was associated with better OS (HR = 0.223, *p* = 0.046) and PFS (HR = 0.244, *p* = 0.011). Patients with grade 2 irLI showed better median OS (not reached) than those without irLI (16.7 months), those with grade 1 (17.5 months), and those with grade ≥ 3 (7.3 months) (overall log-rank *p* = 0.037). Furthermore, patients with grade 2 irLI demonstrated significantly enhanced PFS (not reached) compared to those without irLI (5.7 months), grade 1 (4.6 months), or grade ≥ 3 (2.3 months), with an overall log-rank *p* = 0.010. In addition, patients with grade 2 irLI had the highest disease control rate (overall *p* = 0.053). Conclusion: In patients with uHCC treated with Ate/Bev, moderate elevation of liver enzymes (grade 2 irLI) was associated with significantly improved survival and tumor control.

## Simple Summary

Atezolizumab plus bevacizumab is the standard first-line therapy for unresectable hepatocellular carcinoma (uHCC); however, the impact of immune-related liver injury (irLI) on outcomes remains unclear. This study analyzed 116 patients with uHCC who fulfilled the IMbrave150 criteria, grading irLI using CTCAE v5.0. IrLI occurred in 61 patients (52.6%) with a median onset of 1.7 months. Multivariate analysis showed that ALBI grade II and BCLC stage C predicted poorer survival, whereas grade 2 irLI independently predicted improved outcomes. Patients with grade 2 irLI had the best median OS and PFS compared to those with no irLI, grade 1, or grade ≥ 3 irLI, and they achieved the highest disease control rate. These findings suggest that moderate irLI correlates with significantly better survival and tumor control in uHCC treated with atezolizumab plus bevacizumab.

## 1. Introduction

Hepatocellular carcinoma (HCC) is the leading cause of cancer-related mortality. In recent years, immune checkpoint inhibitors (ICIs) have emerged as promising frontline treatments for unresectable HCC (uHCC). The combination of atezolizumab and bevacizumab (Atezo/Bev) is recommended as the preferred first-line therapy for uHCC in multiple international guidelines [1,2,3,4,5,6,7,8]. However, ICIs are associated with a unique spectrum of immune-related adverse events (irAEs), especially immune-related liver injury (irLI), which can manifest as hepatitis with elevated liver enzymes and/or bilirubin levels, which may lead to liver failure [9,10]. A systematic review by Zheng et al. reported that the incidence of any-grade and high-grade hepatotoxicity in patients with HCC treated with Atezo/Bev ranged from approximately 8–11% and 1–2%, respectively [11].

Mechanistically, ICI-induced liver injury is thought to involve the loss of immune tolerance and T cell-mediated attack on hepatocytes, although the precise mechanisms remain unclear [12]. Several studies have reported an association between the occurrence of irAEs and improved clinical outcomes in patients with cancer receiving ICIs [13]. Nam et al. found that the development of mild (grade 1–2) irAEs was independently associated with improved overall survival (OS) in patients treated with atezo/bev, whereas severe (grade ≥ 3) irAEs were associated with worse outcomes [14]. Similarly, Pinato et al. reported that the occurrence of grade ≥ 2 irAEs was correlated with significantly improved OS in patients with ICI-treated HCC [15]. However, the specific association between irLI and clinical outcomes in patients with uHCC remains unclear. Therefore, we conducted a real-world study to investigate the impact of irLI occurrence and severity on outcomes in uHCC patients receiving atezo/bev, and to assess whether irLI could serve as a prognostic surrogate in this setting.

## 2. Materials and Methods

### 2.1. Patients’ Enrollment

This retrospective study enrolled 235 patients with uHCC who received Ate/Bev therapy at Chang Gung Memorial Hospital, Linkou Medical Center, between September 2020 and August 2023. The inclusion criteria were Barcelona Clinic Liver Cancer (BCLC) stage C or unsuitability for locoregional therapy such as transarterial chemoembolization (TACE) in BCLC stage B, age ≥ 18 years, ECOG performance status ≤ 2, Child–Pugh class A liver function, atezolizumab plus bevacizumab as first-line therapy, and at least two courses of systemic therapy. A total of 116 patients were eligible for analysis in this study (Supplementary Figure S1). This study was approved by the Institutional Review Board of CGMH (IRB 202200991B0C501). Patient consent for participation was not required because of the retrospective nature of this study, which was approved by the Institutional Review Board of CGMH.

### 2.2. Treatment and Follow-Up Protocol

All patients were treated with atezolizumab (1200 mg fixed dose) and bevacizumab (5–15 mg/kg) intravenously every three weeks. Treatment was continued until disease progression, intolerable toxicity, or withdrawal of consent. HCC was diagnosed and treated according to the American Association for the Study of Liver Diseases (AASLD) [2] and European Association for the Study of the Liver and European Organization for Research and Treatment of Cancer (EASL/EORTC) [8] guidelines. Radiological tumor responses were evaluated by two independent radiologists according to the modified Response Evaluation Criteria in Solid Tumors (mRECIST) [16]. The objective response rate (ORR) was assessed as complete response (CR) plus partial response (PR), and the disease control rate (DCR) was assessed as ORR plus stable disease (SD). Imaging studies using dynamic computed tomography (CT) or Magnetic Resonance Imaging (MRI) were monitored every 9–12 weeks and serum AFP levels were measured every three weeks post-therapy. All adverse events (AEs) were evaluated according to the Common Terminology Criteria for Adverse Events v5.0 (NCI CTCAE version 5.0).

### 2.3. Definitions of Immune-Related Liver Injury

IrLI was defined as an increase in serum alanine aminotransferase (ALT) and/or aspartate aminotransferase (AST) levels, deemed to be treatment-related and graded according to the CTCAE v5.0 (grade 1: >upper limit of normal (ULN)-3.0 × ULN if baseline was normal; 1.5–3.0 × baseline if baseline was abnormal, grade 2: >3.0–5.0 × ULN if baseline was normal or abnormal, etc.). In patients with abnormal ALT levels (>1.5× ULN) before the initiation of treatment, irLI was graded according to the severity of baseline derangement, as reported by CTCAE v5.0, after excluding other causes, such as viral infections, active alcohol use, other drugs (e.g., statins and herbal supplements), and disease progression. For HBsAg-positive individuals who did not receive prophylactic nucleos(t)ide analogs before the initiation of therapy, HBV DNA quantification was performed again to exclude HBV reactivation, defined as a >10-fold increase in HBV DNA or new detectability of viral DNA according to the AASLD guidelines [17] if the liver enzyme levels increased. Notably, imaging, including ultrasound, CT, and MRI, to rule out biliary obstruction or HCC progression, was routinely performed in all cases with elevated liver enzyme levels. Patients with a history of autoimmune hepatitis were excluded from atezo/beva therapy according to the HCC guidelines [2,8]. For patients without such a history, if liver enzyme levels were elevated and autoimmune markers (e.g., antinuclear antibodies and anti-smooth muscle antibody) were also elevated, the increase in liver enzymes was considered unrelated to irLI and was excluded [18]. Furthermore, to accurately determine liver injury, our multidisciplinary team, including hepatologists, oncologists, and radiologists, retrospectively reviewed each ALT/AST elevation. If enzyme spikes closely followed an LRT procedure (e.g., within 1–2 weeks) with imaging evidence of post-embolization necrosis, we considered the contribution of LRT to the enzyme spike. Otherwise, elevations occurring more remotely from LRT were attributed to immunotherapy after excluding tumor progression and other causes.

### 2.4. Statistical Analysis

Demographic data and clinical characteristics are presented as numbers with percentages for categorical variables and medians with interquartile ranges (IQR] for continuous variables. The baseline characteristics of each group were compared using the Kruskal–Wallis test, followed by Bonferroni post hoc multiple comparisons. Categorical variables were compared using Pearson’s χ^2^ test or Fisher’s exact test. Stepwise Cox regression models were used to determine the predictors of overall survival (OS) and progression-free survival (PFS). Univariate analysis was performed to evaluate possible prognostic factors, and variables with *p* < 0.05 in the univariate analyses were entered into multivariate analysis performed by Cox proportional hazard regression with hazard ratios (HRs) and 95% confidence intervals (95% CIs). PFS was defined as the time from the date of the first administration of atezolizumab plus bevacizumab to radiological disease progression or death, whichever occurred first. Patients were censored at the date of the last contact or data cutoff for patients who were still alive without radiologically confirmed disease progression. OS was calculated from the start of atezolizumab plus bevacizumab treatment to the date of death. Survival analysis was performed using the Kaplan–Meier method, and subgroups were compared using the log-rank test. Statistical significance was determined as a 2-tailed *p* value < 0.05. All data analyses were performed using the SPSS version 29 (IBM SPSS Inc., Armonk, NY, USA) statistical software package.

## 3. Results

### 3.1. Clinical Characteristics of Enrolled Patients

There was a total of 116 patients with uHCC who received first-line atezo/bev and met the inclusion criteria (Table 1). The median age was 64 years (interquartile range [IQR], 58–72 years), and the majority were male (78.4%). Chronic hepatitis B virus (HBV) infection was the most common underlying etiology (62.9% of patients), followed by hepatitis C virus (HCV) infection (19.0%). Nearly half of the patients (49.1%) had ALBI grade 1 liver function at baseline, and 19.8% had esophageal varices. In terms of tumor burden, the median diameter of the largest target lesion was 9.1 cm (IQR 4.6–11.9). Over half of the patients (56.0%) had more than three tumor nodules, 51.7% had portal vein thrombosis (PVT), 37.1% had extrahepatic spread (EHS), and 73.3% were categorized as BCLC stage C at the initiation of therapy. Atezo/Bev was used in BCLC-B patients typically when high tumor burden with beyond up-to-seven that were unsuitable for transarterial chemoembolization (TACE) (*n* = 24) or refractory to TACE therapy after several times treatment (*n* = 20). Forty-nine patients (42.2%) received combined locoregional therapy (LRT); the majority received radiotherapy (*n* = 44) for portal vein thrombosis, while the others received TACE for small tumors. The median number of atezo/bev cycles delivered was 4 (IQR 3–7), and the median follow-up duration was 9.8 months.

IrLI of any grade occurred in 61 patients (52.6%), typically early during treatment (median onset, 1.7 months). This included eight patients (6.9%) who developed grade ≥ 3 elevations in ALT/AST (more than 5× ULN), consistent with the ~5% incidence of significant hepatotoxicity reported in the IMbrave150 trial. There was no statistically significant difference in the median onset of irLI between patients with and without LRT (1.5 vs. 1.9 months, *p* = 0.336). Compared with patients who did not develop irLI, those who did were more often male (86.9% vs. 69.1%, *p* = 0.020) and had lower use of HBV prophylaxis (26.5% vs. 43.5% received antiviral therapy, *p* = 0.043). They also tended to have a higher baseline tumor burden: patients with irLI had larger tumors (median 9.7 cm vs. 7.9 cm, *p* = 0.011), more frequently had ≥3 tumor nodules (68.9% vs. 41.8%, *p* = 0.014), and were more likely to exceed the “up-to-seven” criteria (88.5% vs. 69.1%, *p* = 0.010). Additionally, patients who developed irLI had higher baseline platelet counts (median 195 vs. 168 × 103/µL, *p* = 0.038) and experienced a shorter median duration of atezo/bev therapy (2.3 months) than those without irLI (2.8 months, *p* = 0.038), suggesting that liver toxicity led to more frequent treatment interruptions or early discontinuation of therapy.

### 3.2. Baseline Characteristics by irLI Severity

Among the 61 patients who experienced irLI, we further stratified the liver injury severity as grade 1 (mild, *n* = 37), grade 2 (moderate, *n* = 14), or grade ≥ 3 (severe, *n* = 8). The baseline characteristics of the subgroups are presented in Table 2. There were no significant differences among the three groups in terms of age or sex distribution, viral etiology (HBV vs. HCV), baseline ALBI grade, or major tumor characteristics (tumor size, tumor number, presence of EHS, and overall tumor stage), except for differences in PVT incidence. Notably, patients who developed grade 2 irLI had distinct features compared to those with mild or severe irLI. The grade 2 group had the lowest prevalence of baseline liver inflammation; only 64.3% had any ALT/AST elevation prior to therapy, compared with 89.5% of grade 1 patients and 100% of grade ≥ 3 patients (*p* = 0.035). They also had the lowest incidence of baseline PVT (28.6% in grade 2 vs. 66.7% in grade 1 and 50.0% in grade ≥ 3; *p* = 0.045) and the smallest proportion of tumors exceeding the up-to-seven criteria (64.3% vs. 94.9% and 100.0% in grades 1 and ≥3, respectively; *p* = 0.005). Conversely, patients in the grade 2 subgroup were the most likely to receive combination locoregional therapy with atezo/bev (57.1% vs. 41.0% of grade 1 and 50.0% of grade ≥ 3; *p* = 0.046). They also remained on Atezo/Bev treatment longer, with a median of 6.7 months of therapy for grade 2 patients, compared to 3.2 months for grade 1 and 2.2 months for grade ≥ 3 (*p* = 0.042). Reflecting a more moderate toxicity profile, only 14.3% of patients with grade 2 irLI required corticosteroids to manage liver toxicity, whereas 20.5% of patients with grade 1 and 50.0% of patients with grade ≥ 3 irLI received steroids (*p* = 0.043). Prophylactic antiviral use was more common in the grade 2 group (40.0%) than in the grade 1 (30.3%) or grade ≥ 3 (0%) groups, although this trend was not statistically significant (*p* = 0.061). These differences suggest that patients who experienced only moderate liver injury tended to have better baseline liver reserves and were more often maintained on therapy, potentially contributing to improved outcomes.

### 3.3. Treatment Responses and Survival Outcomes

The treatment responses to Ate/Bev are summarized in Supplementary Table S1. At the first on-treatment imaging evaluation, three patients (2.6%) achieved a complete response (CR), and 38 (32.8%) achieved a partial response (PR), yielding an initial ORR of 35.4%. An additional 40 patients (34.5%) had stable disease, with an initial DCR of 69.9%. Over the course of therapy, some patients showed improved responses; ultimately, the best overall responses observed were CR in nine patients (7.8%) and PR in 37 patients (31.9%), with an ORR of 39.7%. The best disease stabilization was observed in 36 patients (31.0%), resulting in an overall DCR of 70.7%.

During the follow-up period (median 9.8), tumor progression occurred in 61 patients (52.6%), and 48 patients (41.4%) died at the time of analysis. The median OS for the entire cohort was 27.5 months (95% CI 8.7–46.3), and the median PFS was 5.4 months (95% CI 4.2–6.6) (Supplementary Figure S2). We analyzed various baseline and treatment-related factors associated with the survival outcomes. Univariate analysis revealed that factors significantly associated with shorter OS included ALBI grade II liver function, BCLC stage C, tumor numbers ≥ 3, and the presence of portal vein thrombosis. In contrast, prior locoregional therapy and the occurrence of grade 2 irLI during treatment were associated with longer OS in the univariate analysis. All variables were included in the multivariate Cox model. In the multivariate analysis (Table 3), three factors remained independent predictors of OS: ALBI grade II (vs. grade I) liver function (HR 2.003, 95% CI, 1.079–3.720; *p* = 0.028) and BCLC stage C (vs. stage A/B) (HR 3.876, 95% CI, 1.288–11.66; *p* = 0.016) were associated with significantly worse survival, whereas the occurrence of grade 2 irLI was associated with significantly improved survival (HR 0.223, 95% CI 0.051–0.974; *p* = 0.046). Similarly, for PFS, univariate analysis indicated that age ≥ 65 years, ALBI grade II, BCLC stage C, tumor numbers ≥ 3, AFP ≥400 ng/mL, prior LRT, and occurrence of grade 2 irLI were significant factors. Multivariate Cox analysis for PFS identified three independent prognostic factors: ALBI grade II liver function (HR 1.327, 95% CI 1.063–2.308; *p* = 0.046) and BCLC stage C (HR 1.790, 95% CI 1.115–3.503; *p* = 0.039) were associated with shorter PFS, whereas grade 2 irLI was associated with prolonged PFS (HR 0.244, 95% CI 0.082–0.727; *p* = 0.011) (Table 4).

### 3.4. Subgroup Analysis of Patients with irLI Severity

**Figure 1:**
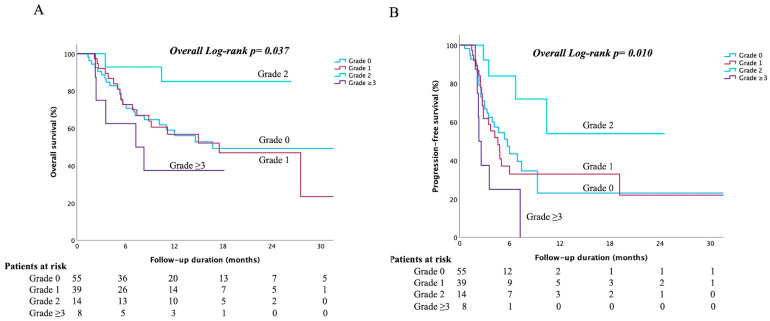
Kaplan–Meier survival curves stratified by the maximum grade of immune-related liver injury (irLI). (**A**) Overall survival by maximum irLI grade (*n* = 55 for grade 0, *n* = 39 for grade 1, *n* = 14 for grade 2, and *n* = 8 for grade ≥ 3): patients with grade 2 (moderate) irLI had the longest median OS, whereas those with grade ≥ 3 irLI had the shortest OS. (**B**) Progression-free survival (PFS) by maximum irLI grade: Patients with grade 2 irLI had the longest PFS, whereas those with grade ≥ 3 irLI had the shortest.

We next analyzed the outcomes according to the maximum grade of irLI experienced, including patients with no irLI (grade 0) for comparison purposes. Patients who developed grade 2 (moderate) irLI had the most favorable survival rates. Their median OS was not reached, versus approximately 16–17 months in those with no irLI or grade 1 irLI and 7.3 months in those with grade ≥ 3 irLI (log-rank *p* = 0.037; Figure 1A). Similarly, the median PFS was not reached for the grade 2 group, compared to 5.7, 4.6, and 2.3 months in the grade 0, 1, and ≥3 groups, respectively (log-rank *p* = 0.010; Figure 1B). Thus, moderate immune-mediated hepatotoxicity was associated with significantly better survival than no, mild, or severe liver injury.

We also explored whether irLI severity correlated with tumor response. Although the ORR and DCR did not differ significantly among the subgroups, they were the highest in the grade 2 irLI group. For example, patients with grade 2 irLIs achieved an ORR of 50.0% and a DCR of 92.9%, compared to 41.8% and 67.3% in patients without irLIs and 25.0% and 50.0% in those with grade ≥ 3 irLIs, respectively. Although these differences were not statistically significant (overall *p* > 0.05), they suggested a trend toward improved tumor control in patients who experienced moderate irLI. The detailed response data for the irLI subgroup are provided in Supplementary Table S2.

### 3.5. Corticosteroid Use and Outcomes

Corticosteroid therapy for irLI (at doses equivalent to ≥10 mg prednisone daily) was administered to 14 of 61 patients (23.0%) who developed irLI, with a median duration of 55 (range 11–157) days, with tapering guided by normalization of ALT/AST. Corticosteroid therapy was administered to 20.5% of grade 1 and 14.3% of grade 2 patients for liver toxicity. The lower steroid rate in grade 2 can reflect clinician judgment: in some grade 1 cases with rising AST/ALT (near the grade 2 threshold) or other risk factors, steroids were administered preemptively, whereas in some grade 2 cases, the clinicians observed a spontaneous fall. Among those who experienced severe irLI (grade ≥ 3), 4 four of eight patients (50%) received steroid treatment to manage the event (the remainder did not, often due to clinical contraindications or physician judgment). Notably, two patients with grade ≥ 3 irLI died during the liver injury episode; neither had received timely steroid intervention because of concerns regarding potential hepatitis B reactivation or serious infection. Therefore, steroid use was not dictated by a strict protocol but rather by clinical concerns. We compared the outcomes between patients who did and did not receive steroids for irLI. Paradoxically, patients who required steroid therapy had a shorter median OS (11.2 months) than those who did not receive steroids (27.5 months), although this difference was not statistically significant (log-rank *p* = 0.574). The median PFS was also similar between the steroid-treated and non-steroid-treated patients (4.8 vs. 5.0 months, *p* = 0.324) (see Supplementary Figure S3). These observations suggest that the need for steroids (often reflecting more severe toxicity) may be associated with poorer outcomes, although the data were not statistically significant.

To further dissect the impact of steroids, we performed subgroup analyses based on irLI severity. Among patients with grade 1 irLI, there were no significant differences in OS or PFS between those who received steroids and those who did not (log-rank *p* = 0.451 and *p* = 0.961, respectively) (Supplementary Figure S4A,B). In the subset of patients with grade ≥ 2 irLI, there was a trend toward improved OS in those who received steroids (median OS not reached with steroids vs. 16.2 months without steroids), but this did not reach statistical significance given the small sample size (log-rank *p* = 0.093; Supplementary Figure S4C). There was no apparent difference in PFS between the grade ≥ 2 groups with or without steroid use (*p* = 0.421; Supplementary Figure S4D). In summary, corticosteroid therapy was reserved primarily for severe irLI cases in this cohort, and although it may have provided some benefit in the most severe cases, steroid use did not significantly improve outcomes in patients with mild irLI.

### 3.6. The Factors Determined Occurrence of Grade 2 irLI

Given that patients with grade 2 irLI showed the most favorable outcomes, we sought to identify the factors associated with the occurrence of grade 2 irLI. This analysis was confined to 61 patients who developed any irLI, compared to those who had grade 2 irLI (*n* = 14) and those with grade 1 or ≥3 irLI. In the univariate analysis, several factors were associated with a higher likelihood of experiencing grade 2 (as opposed to non-grade 2) irLI: receiving antiviral therapy, having normal baseline liver enzyme levels (ALT/AST within the ULN), and undergoing combination locoregional therapy were all positively associated with grade 2 irLI occurrence. In the multivariate analysis (Supplementary Table S3), two factors emerged as independent predictors of grade 2 irLI. Antiviral therapy was protective against severe liver injury and corresponded to increased odds of only moderate irLI (HR 0.726, *p* = 0.043 for grade 2 vs. others). Additionally, a combination of LRT and atezo/bev was independently associated with the occurrence of grade 2 irLI (HR 1.230, *p* = 0.045). Among the patients who received combined LRT, most underwent radiotherapy (six patients, 42.9% of those with combination therapy), and a smaller proportion underwent TACE (two patients, 14.3%). These findings suggest that prophylactic antiviral treatment and the proinflammatory stimulus of locoregional therapies might tilt the balance toward moderate immune-mediated liver inflammation (as opposed to no or overly severe inflammation) during ICI therapy. Subgroup analyses were performed to assess their independent effects and clarify whether these variables influenced survival outcomes. We further stratified patients who received LRT and found that the survival advantage associated with grade 2 irLI was present in patients with and without LRT (Supplementary Figure S5A,C), and the difference was statistically significant in PFS for those with LRT (Supplementary Figure S5B) and marginally significant for those without LRT (Supplementary Figure S5D). When stratified by antiviral prophylaxis status, the association between moderate irLI and survival was not observed in patients receiving antiviral therapy (Supplementary Figure S6A,B); however, the survival advantage associated with grade 2 irLI was present in patients without antiviral therapy (Supplementary Figure S6C,D).

**Figure 2:**
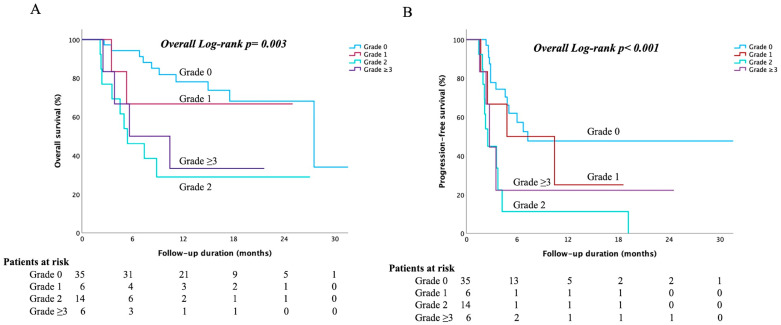
Kaplan–Meier survival curves according to the grade of immune-related “icteric” hepatitis (immune-mediated hyperbilirubinemia) among patients who developed irLI. (**A**) OS according to bilirubin grade *(n* = 35 for grade 0, *n* = 6 for grade 1, *n* = 14 for grade 2, *n* = 6 for grade ≥ 3): patients with grade ≥ 2 bilirubin elevation had significantly worse OS than those with grade 0–1 bilirubin elevation. (**B**) PFS by bilirubin grade: patients with grade ≥ 2 immune-related hyperbilirubinemia had significantly worse PFS than those with grades 0–1.

Further analysis was performed to evaluate the effects of immune-mediated icteric hepatitis (irLI concurrent with elevated bilirubin levels). Among patients who developed irLI, we compared outcomes based on the grade of total bilirubin elevation (grade 0, no significant bilirubin rise; grade ≥ 3, severe hyperbilirubinemia). Patients who experienced grade 2 or higher bilirubin elevation (i.e., clinically apparent immune-related hepatitis with jaundice) had markedly worse survival outcomes. In the subset of patients with irLI, the median OS for those with grade 0, 1, 2, and ≥3 bilirubin elevation was 27.5 months, not reached, 5.4 months, and 5.6 months, respectively (overall log-rank *p* = 0.003; Figure 2A). Similarly, the median PFS was 7.3, 4.8, 2.5, and 2.7 months for bilirubin grades 0, 1, 2, and ≥3, respectively (overall *p* < 0.001; Figure 2B). Thus, while a moderate increase in liver enzymes alone (grade 2 irLI) was associated with better outcomes, the development of immune-related icterus (even grade 2 bilirubin elevation) signaled a poor prognosis akin to severe liver injury. This dichotomy underscores the fact that irLI confined to liver enzymes is very different from irLI, which affects liver function (bilirubin), with the latter being much more detrimental to the patient.

### 3.7. Other Immune-Related Adverse Events

In addition to liver toxicity, a wide array of other irAEs were observed (Supplementary Table S4). Eighty patients (69.0%) experienced at least one irAE outside the liver, and 18 (15.5%) had severe irAEs (grade ≥ 3) affecting other organ systems. The first irAE typically occurred early (median onset, 1.3 months into therapy). Patients with more severe irLIs tended to have higher rates of severe irAEs in other organs. For example, grade ≥ 3 extrahepatic irAEs occurred in 21.4% of patients with grade 2 irLI and 37.5% of those with grade ≥ 3 irLI, compared to ~7% in patients with no or grade 1 irLI.

The most common non-hepatic irAEs of any grade were thrombocytopenia (28.4%), proteinuria (24.1%), thyroid dysfunction (19.8%; primarily hypothyroidism), and hypertension (18.1%). Notably, significant gastrointestinal bleeding occurred in 8.6% of patients (which may have been related to bevacizumab or underlying cirrhosis rather than an immune-mediated process). Dermatologic irAEs, such as rashes, were relatively uncommon (1.7%). Overall, these data underscore that while liver injury was the predominant toxicity of Atezo/Bev in HCC, other immune toxicities were also frequent and should be closely monitored. Moreover, the tendency for severe irAEs to co-occur in patients with severe irLI suggests a globally heightened immune reactivity in these individuals.

### 3.8. Comparison of Survival and Efficacy After Propensity Score Matching

Patients with moderate irLI (grade 2) had better baseline features than those with other grades. To minimize the influence of confounding factors, propensity score matching (PSM) was performed using the following variables: ALBI score, tumor burden, and use of LRT (Supplementary Table S5). After 1:1:1:1 matching for the four groups (irLI grades 0, 1, 2, and ≥3), only eight patients from each group were analyzed because of the small sample size. No significant differences in the baseline characteristics were observed among the four groups. Patients with grade 2 irLI had a trend of better initial and better ORR, although not reaching statistical significance, and better DCR than other patients (Supplementary Table S6). Univariate Cox analysis showed that grade 2 irLI was the only predictor of OS (HR 0.221, 95% CI 0.028–0.933; *p* = 0.041; Supplementary Table S7) and PFS (HR 0.208, 95% CI 0.047–0.915; *p* = 0.038; Supplementary Table S8). Patients who developed grade 2 irLI had longer OS (irLI grade 0 vs. 1 vs. 2 vs. ≥3:16.7 vs. 15.0 vs. not reached vs. 7.3 months, overall log-rank *p* = 0.017; Supplementary Figure S7A) and PFS (irLI grade 0 vs. 1 vs. 2 vs. ≥3:2.6 vs. 10.4 vs. 19.2 vs. 3.3 months, overall log-rank *p* < 0.001; Supplementary Figure S7B) than patients with other grade irLI.

## 4. Discussion

Patients undergoing ICI therapy for HCC frequently experience immune-mediated hepatic adverse events [11]. Severe irLI can necessitate treatment interruption or discontinuation and often requires high-dose corticosteroids, which carry the risk of metabolic complications and serious infection. In our study, irLI occurred in 52.6% of uHCC patients treated with atezo/bev, which is higher than the incidence reported in the pivotal IMbrave150 trial (~8–11%) [19] but aligns with other real-world cohorts [20]. This discrepancy likely reflects our patient population, as the majority had HBV-related HCC, which may be predisposed to hepatic immune flares during ICI therapy. The median time to onset of irLI was 1.7 months, consistent with prior observations that irAEs often manifest early during ICI treatment [21,22]. To the best of our knowledge, this study is the first to demonstrate that HCC patients who develop moderate immune-related hepatitis (CTCAE grade 2), as opposed to no, mild, or severe liver injury, achieve the most favorable outcomes with atezo/bev therapy. In our cohort, grade 2 irLI was independently associated with prolonged OS and PFS, as well as higher tumor response and disease control rates. In contrast, patients with severe liver injury (especially those with immune-related hyperbilirubinemia) had the poorest outcomes. These findings suggest that a moderate immune reaction in the liver may reflect or facilitate a more effective antitumor immune response, whereas an overly severe reaction may be detrimental, potentially due to premature treatment cessation or direct organ damage.

Previous studies [23] have also reported that the development of a hand–foot skin reaction (HFSR) is associated with improved PFS, which is closely attributed to cumulative doses of sorafenib in HCC patients. Importantly, the association between ICI treatment duration and OS and PFS only showed marginal significance in the univariate analysis. Moreover, no statistically significant association was found between ICI treatment duration and the occurrence of grade 2 IrLI. This study aimed to ensure that the survival benefit associated with grade 2 irLI was not merely due to prolonged therapy duration.

Our results add to the growing body of evidence linking irAEs to improved cancer outcomes in HCC. Pinato et al. and others reported that patients who experience treatment-related toxicities, particularly grade ≥ 2 irAEs, tend to have better treatment efficacy with ICIs. In contrast, a recent multicenter study found that patients who developed severe irAEs (grade ≥ 3) had worse survival, likely due to therapy discontinuation and organ dysfunction. A global analysis focusing specifically on ICI-related liver injury showed that patients with HCC and grade 1–2 irLI had improved OS compared to those without irLI; however, patients who developed hepatic decompensation (liver failure) within 6 months of starting atezo/bev had significantly higher early mortality. Taken together, these observations imply that moderate immune-mediated hepatic inflammation, when not accompanied by liver decompensation, may serve as a surrogate marker of effective antitumor immunity, whereas severe liver toxicity is associated with poor outcomes. Consistently, in our study, patients with grade 2 irLI (without significant bilirubin elevation) had the longest survival, while those with grade ≥ 2 “icteric” irLI (with jaundice) had the shortest survival duration. Overall, our findings align with previously published observations in both the immunotherapy [24] and tyrosine kinase inhibitor [24,25] eras [15,25,26], which demonstrated a positive association between the development of certain treatment-related toxicities and improved outcomes in HCC.

The positive correlation between moderate hepatic toxicity and favorable outcomes in our study raises important questions regarding the possible underlying mechanisms of this association. One hypothesis is that irLI reflects robust activation of the immune system by therapy; in other words, patients who mount an immune response vigorous enough to inflame the liver might also mount an especially effective immune attack on their cancer [13,14]. In this scenario, liver injury is essentially a byproduct of a heightened immune state that eradicating tumor cells. An alternative (but not mutually exclusive) hypothesis is that liver injury itself contributes to antitumor immunity, for instance, through the release of tumor antigens or “danger signals” from damaged hepatocytes that further stimulate immune responses [9]. Several observations in our study support these findings. We found that patients with grade 2 irLI had a significantly higher incidence of concurrent locoregional therapy and antiviral prophylactic interventions, which can modulate the immune milieu. Locoregional treatments, such as radiotherapy, can amplify systemic immune activation by inducing immunogenic tumor cell death and altering the tumor microenvironment. Indeed, combined radiotherapy was administered more frequently to patients who developed grade 2 irLI, and combination LRT emerged as an independent predictor of moderate liver injury in our study. For example, our group observed that adding high-dose radiotherapy to atezo/bev can augment efficacy in advanced HCC (presumably via an abscopal effect) [27]. Radiotherapy has been shown to increase intratumoral CD8 + T-cell infiltration in HCC [28], and TACE can induce immunogenic cell death, boosting antitumor immunity [29], thus supporting a synergistic effect between locoregional therapy and immunotherapy [30]. In our study, the association of LRT with moderate (rather than severe) irLI implies that such immune priming yields a controlled, effective immune response instead of an overwhelming response.

Another notable factor influencing hepatotoxicity was the underlying liver condition and viral prophylaxis. Patients with HCC often have chronic HBV or HCV infections, which can flare during ICI therapy if not suppressed prior to treatment. Hung et al. [20] reported that abnormal baseline liver function (often reflecting active hepatitis or advanced cirrhosis) is an independent risk factor for hepatitis flare-ups during immunotherapy. In our cohort, baseline liver enzyme abnormalities did not differ significantly between patients who did and did not develop irLI, likely because all patients had preserved liver function (Child–Pugh A) at baseline. However, none of the patients who experienced grade ≥ 3 irLI were on antiviral prophylaxis, and all had some baseline liver dysfunction and high tumor burden, a combination that may predispose them to severe hepatic decompensation upon immune stimulation. In contrast, patients with grade 2 irLI had the lowest rate of baseline ALT/AST elevation, and a substantial proportion were receiving antiviral therapy. Our multivariate analysis suggested that antiviral prophylaxis independently protects against severe liver injury and is associated with an increased likelihood of moderate irLI. This highlights the importance of antiviral prophylaxis in HBV-infected patients to prevent uncontrolled hepatitis during ICI therapy and suggests that maintaining optimal baseline liver health (through disease management and careful patient selection) may allow patients to experience moderate, manageable immune hepatitis rather than a catastrophic one.

Despite the high incidence of irLI in our study, most cases were relatively mild and managed conservatively. Only 23% of the patients required systemic corticosteroid intervention, nearly all of whom had hepatitis grade ≥ 3. Notably, steroid-treated patients in our cohort had shorter survival, likely because steroids were predominantly used in the most severe cases. Clinicians treating HCC are often judicious with corticosteroids due to underlying cirrhosis, and there is concern that immunosuppressive therapy could diminish the antitumor efficacy of ICIs and increase the risk of infection in an already immunocompromised population. Indeed, a nationwide study reported that high-dose corticosteroid use for irAEs in patients with cancer was associated with an increased risk of serious infection [31]. The safety and optimal timing of steroid use for irLI in HCC remain to be defined [9]. Our practice has been to avoid steroids for low-grade (grade 1) liver enzyme elevations, managing these patients with close monitoring and supportive care while excluding other causes of hepatitis, as most mild cases resolve spontaneously. For grade ≥ 2 hepatitis, we initiated corticosteroids to prevent progression to liver failure and observed a trend that steroid use in patients with significant liver injury might improve survival, although this did not reach statistical significance. Overall, our findings support a management strategy of tolerating moderate liver enzyme elevations (to maintain ICI therapy) while promptly treating severe immune-mediated hepatitis, thereby maximizing the oncologic benefits and minimizing the risk of fatal toxicity.

This study had several limitations that should be considered. This was a retrospective, single-center analysis with a small sample size, which may limit the generalizability of our findings to the general population. However, high-grade irLI is uncommon in the IMbrave150 trial; even in this sample, the survival benefit was pronounced and statistically significant, even after propensity score matching for confounders. External validation using large-scale databases, such as the Taiwan National Health Insurance Research Database (NHIRD), is warranted. Although the NHIRD does not contain routine laboratory data to precisely define irLI, surrogate markers (e.g., diagnostic codes for hepatitis and corticosteroid prescriptions after immunotherapy) may serve as proxies for immune-related hepatic injury. Future integration of the NHIRD with cancer registry and laboratory datasets will be essential to validate whether moderate irLI consistently predicts improved outcomes in real-world settings. Second, we defined irLI according to the CTCAE as an elevation in ALT/AST (hepatocellular injury). We did not systematically collect ALK-P or r-GT data for each event because most of the data were lacking or incomplete. However, we evaluated the total bilirubin levels and performed imaging studies to exclude cholangitis or tumor progression, which could lead to duct compression. We also did not perform mechanistic immune studies or liver biopsies, instead relying on clinical criteria for diagnosing irLI. Therefore, our inference that moderate ALT/AST elevation reflects a more vigorous antitumor immune response is indirect, and some cases of liver injury may have been misclassified.

Despite these caveats, our real-world data provide insights into the spectrum of immunotherapy-related liver injury in HCC and its prognostic significance. These observations generate hypotheses that should be validated prospectively and mechanistically investigated in future studies with larger sample sizes.

## 5. Conclusions

For patients with uHCC receiving atezolizumab plus bevacizumab, the development of moderate immune-related liver injury (grade 2 elevation of liver enzyme levels) was independently associated with improved survival and tumor response, suggesting that moderate irLI is a positive prognostic indicator. Integrative management strategies, such as antiviral prophylaxis and combination locoregional therapy, may help foster this “optimal” immune response by protecting the liver from extreme injury while enhancing antitumor immunity. However, severe irLI, particularly when accompanied by hyperbilirubinemia, leads to poor outcomes and should be prevented. Most cases of irLI can be managed conservatively, and corticosteroids should be reserved for high-grade hepatitis because their benefits in less severe cases are unclear. Future studies should aim to confirm these findings in larger cohorts and elucidate the immunological mechanisms linking irLI to antitumor efficacy. A better understanding of this relationship could aid clinicians in predicting patient outcomes and tailoring immunotherapy for HCC by striking an appropriate balance between the therapeutic benefits and toxicity.

## Figures and Tables

**Table 1:** Baseline characteristics of the enrolled patients.

Variables	Overall (*n* = 116)	No irLI (*n* = 55)	Any Grade irLI (*n* = 61)	*p* Value
Age (year-old)	64.1 (IQR 57.7–71.9)	64.5 (IQR 57.8–72.3)	64.0 (IQR 56.4–71.5)	0.831
Male gender, n (%)	91 (78.4)	38 (69.1)	53 (86.9)	0.020
HBV/HCV/NBNC, *n* (%)	73/22/21 (62.9/19.0/18.1)	33/13/9 (60.0/23.6/16.4)	40/9/12 (65.6/14.8/19.7)	0.467
Antiviral therapy, *n* (%)	33/95 (34.7)	20/46 (43.5)	13/49 (26.5)	0.043
NLR	3.40 (IQR 2.51–4.99)	3.38 (IQR 2.28–4.66)	3.49 (IQR 2.70–5.99)	0.308
AST (IU/mL)	53 (IQR 36–82)	54 (IQR 32–91)	51 (IQR 37–82)	0.936
ALT (IU/mL)	48 (IQR 30–73)	50 (IQR 31–99)	46 (IQR 27–68)	0.180
Baseline AST or ALT > ULN, *n* (%)	97 (83.6)	46 (83.6)	51 (83.6)	0.811
Bilirubin total (mg/dL)	0.80 (IQR 0.50–1.20)	0.85 (IQR 0.50–1.28)	0.80 (IQR 0.50–1.15)	0.593
Albumin (g/dL)	3.78 (IQR 3.40–4.16)	3.79 (IQR 3.29–4.15)	3.76 (IQR 3.55–4.19)	0.519
Platelet (10^3^/μL)	188 (IQR 145–284)	168 (IQR 134–245)	195 (IQR 153–310)	0.038
ALBI grade I/II, n (%)	57/59 (49.1/50.9)	29/26 (52.7/47.3)	28/33 (45.9/54.1)	0.463
Portal vein thrombosis, *n* (%)	60 (51.7)	26 (47.3)	34 (55.7)	0.362
Esophageal varices, *n* (%)	23 (19.8)	10 (18.2)	23 (21.3)	0.673
Extrahepatic metastasis, *n* (%)	43 (37.1)	19 (34.5)	24 (39.3)	0.593
BCLC stage B/C, *n* (%)	31/85 (26.7/73.3)	17/38 (30.9/69.1)	14/47 (23.0/77.0)	0.333
Target tumor size, cm	9.1 (IQR 4.6–11.9)	7.9 (IQR 3.9–10.5)	9.7 (IQR 5.7–13.0)	0.011
Tumor number 1/2/ ≥ 3	35/16/65 (30.2/13.8/56.0)	22/10/23 (40.0/18.2/41.8)	13/6/42 (21.3/9.8/68.9)	0.014
Beyond up-to-seven, *n* (%)	92 (79.3)	38 (69.1)	54 (88.5)	0.010
AFP (ng/mL)	377 (IQR 23–5719)	423 (IQR 16–11903)	239 (IQR 28–3307)	0.770
Combination with LRT, *n* (%)	49 (42.2)	21 (38.2)	28 (45.9)	0.401
Prior LRT, *n* (%)	48 (41.4)	25 (45.5)	23 (37.7)	0.397
ICI treatment duration (months)	2.5 (IQR 1.5–4.6)	2.8 (IQR 1.4–4.7)	2.3 (IQR 1.8–6.2)	0.038

Abbreviations: AFP, Alpha-fetoprotein; ALT, alanine aminotransferase; AST, aspartate aminotransferase; BCLC, Barcelona Clinic Liver Cancer classification; HBV, hepatitis B virus; irLI, immune-related liver injury; LRT, locoregional therapy; NLR, Neutrophil-to-lymphocyte ratio; ULN, upper limit of normal.

**Table 2:** Baseline characteristics of patients with irLI.

Variables	Mild, Grade 1 (*n* = 39)	Moderate, Grade 2 (*n* = 14)	Severe, Grade ≥ 3 (*n* = 8)	*p* Value
Age (year-old)	64.0 (IQR 59.3–71.6)	69.1 (IQR 57.3–74.2)	58.4 (IQR 42.3–69.5)	0.389
Male gender, n (%)	32 (82.1)	13 (92.9)	8 (100)	0.294
HBV/HCV/NBNC, *n* (%)	26/7/6 (66.7/17.9/15.4)	9/1/4 (64.3/7.1/28.6)	5/1/2 (62.5/12.5/25.0)	0.752
Antiviral therapy, *n* (%)	10/33 (30.3)	3/10 (30.0)	0/6 (0)	0.061
NLR	3.40 (IQR 2.76–5.91)	3.13 (IQR 2.26–6.02)	3.92 (IQR 2.49–7.89)	0.890
AST (IU/mL)	57 (IQR 42–85)	37 (IQR 29–55)	60 (IQR 45–110)	0.151
ALT (IU/mL)	49 (IQR 30–68)	27 (IQR 21–43)	56 (IQR 34–105)	0.174
Baseline AST or ALT > ULN, *n* (%)	34 (89.5)	9 (64.3)	8 (100)	0.035
Bilirubin total (mg/dL)	0.90 (IQR 0.60–1.30)	0.55 (IQR 0.48–0.80)	0.85 (IQR 0.53–1.50)	0.368
Albumin (g/dL)	3.78 (IQR 3.49–4.22)	3.88 (IQR 3.61–4.17)	3.55 (IQR 2.94–3.96)	0.193
Platelet (10^3^/μL)	192 (IQR 159–295)	249 (IQR 147–315)	262 (IQR 147–361)	0.803
ALBI grade I/II, n (%)	17/22 (43.6/56.4)	9/5 (64.3/35.7)	2/6 (25.0/75.0)	0.183
Portal vein thrombosis, *n* (%)	26 (66.7)	4 (28.6)	4 (50.0)	0.045
Esophageal varices, *n* (%)	9 (23.1)	2 (14.3)	2 (25.0)	0.760
Extrahepatic metastasis, *n* (%)	18 (46.2)	4 (28.6)	2 (25.0)	0.345
BCLC stage B/C, *n* (%)	6/33 (15.4/84.6)	6/8 (42.9/57.1)	2/6 (25.0/75.0)	0.110
Target tumor size, cm	10.4 (IQR 6.7–13.0)	7.8 (IQR 2.4–12.8)	8.9 (IQR 7.5–9.9)	0.304
Tumor number 1/2/ ≥ 3	9/3/27 (23.1/7.7/69.2)	4/2/8 (28.6/14.3/57.1)	0/1/7 (0/12.5/87.5)	0.507
Beyond up-to-seven, *n* (%)	37 (94.9)	9 (64.3)	8 (100)	0.005
AFP (ng/mL)	584 (IQR 46–3961)	76 (IQR 14–363)	280 (IQR 50–3108)	0.669
Combination with LRT, *n* (%)	16 (41.0)	8 (57.1)	4 (50.0)	0.046
Prior LRT, *n* (%)	10 (25.6)	8 (57.1)	5 (62.5)	0.034
ICI treatment duration (months)	3.2 (IQR 1.2–4.8)	6.7 (IQR 2.1–10.3)	2.2 (IQR 1.7–4.6)	0.042
Corticosteroid therapy, *n* (%)	8 (20.5)	2 (14.3)	4 (50.0)	0.043

Abbreviations: AFP, Alpha-fetoprotein; ALT, alanine aminotransferase; AST, aspartate aminotransferase; BCLC, Barcelona Clinic Liver Cancer classification; HBV, hepatitis B virus; irLI, immune-related liver injury; LRT, locoregional therapy; NLR, Neutrophil-to-lymphocyte ratio; ULN, upper limit of normal.

**Table 3:** Predictors of overall survival.

Variables	Univariate			Multivariate		
	HR	95%CI	*p* Value	HR	95%CI	*p* Value
Age ≥ 65 years old (vs. <65 years old)	0.626	0.346–1.132	0.121			
Male (vs. female)	0.604	0.318–1.146	0.123			
Viral infection (vs. others)	1.338	0.600–2.984	0.477			
Antiviral therapy (vs. no)	0.878	0.458–1.684	0.696			
ALBI grade II (vs. I)	2.489	1.381–4.487	0.002	2.003	1.079–3.720	0.028
Baseline AST or ALT > ULN (vs. No)	1.315	0.589–2.938	0.504			
BCLC stage C (vs. stage A/B)	4.137	1.634–10.47	0.003	3.876	1.288–11.66	0.016
Tumor size	1.027	0.967–1.091	0.386			
Tumor numbers ≥ 3 (vs. <3)	2.354	1.283–4.320	0.006	2.379	0.984–4.781	0.075
Portal vein thrombosis (vs. No)	2.229	1.207–4.118	0.010	0.910	0.438–1.889	0.800
Extrahepatic metastasis (vs. No)	1.483	0.838–2.625	0.177			
AFP ≥ 400 ng/mL (vs. <400 ng/mL)	1.249	0.702–2.219	0.449			
Prior LRT (vs. No)	0.477	0.256–0.889	0.020	0.733	0.369–1.454	0.374
Combination with LRT (vs. No)	1.195	0.870–2.129	0.246			
IrLI (vs. No)	0.227	0.055–0.939	0.041			
Grade 1	1.228	0.684–2.203	0.492			
Grade 2	0.227	0.055–0.939	0.041	0.223	0.051–0.974	0.046
Grade ≥ 3	1.964	0.776–4.974	0.155			
Corticosteroid therapy (vs. No)	0.878	0.347–2.220	0.783			
ICI treatment duration	0.870	0.782–1.167	0.097			

Abbreviations: AFP, Alpha-fetoprotein; ALT, alanine aminotransferase; AST, aspartate aminotransferase; LRT, locoregional therapy; NLR, Neutrophil-to-lymphocyte ratio; ULN, upper limit of normal; IrLI, immune-related liver injury.

**Table 4:** Predictors of progression-free survival.

Variables	Univariate			Multivariate		
	HR	95%CI	*p* Value	HR	95%CI	*p* Value
Age ≥ 65 years old (vs. <65 years old)	0.545	0.323–0.919	0.023	0.553	0.310–1.089	0.076
Male (vs. female)	0.939	0.517–1.708	0.837			
Viral infection (vs. others)	1.439	0.682–3.035	0.340			
Antiviral therapy (vs. no)	0.591	0.327–1.068	0.081			
ALBI grade II (vs. I)	1.765	1.059–2.943	0.029	1.327	1.063–2.308	0.046
Baseline AST or ALT > ULN (vs. No)	1.662	0.786–3.515	0.184			
BCLC stage C (vs. A/B)	1.592	12845–2.999	0.040	1.790	1.115–3.503	0.039
Tumor size	1.011	0.959–1.065	0.690			
Tumor numbers ≥ 3 (vs. <3)	3.529	1.981–6.286	0.001	4.579	0.997–9.128	0.061
Portal vein thrombosis (vs. No)	1.324	0.795–2.204	0.281			
Extrahepatic metastasis (vs. No)	1.193	0.713–1.997	0.502			
AFP ≥ 400 ng/mL (vs. <400 ng/mL)	1.759	1.053–2.936	0.031	1.017	0.583–1.775	0.953
Prior LRT (vs. No)	0.496	0.285–0.862	0.013	0.716	0.390–1.314	0.281
Combination with LRT (vs. No)	0.742	0.560–1.385	0.823			
IrLI (vs. No)						
Grade 1	1.153	0.682–1.949	0.596			
Grade 2	0.319	0.115–0.884	0.028	0.244	0.082–0.727	0.011
Grade ≥ 3	2.755	0.943–6.108	0.063			
Corticosteroid therapy (vs. No)	0.930	0.422–2.049	0.857			
ICI treatment duration	0.755	0.667–1.055	0.081			

Abbreviations: AFP, Alpha-fetoprotein; ALT, alanine aminotransferase; AST, aspartate aminotransferase; NLR, Neutrophil-to-lymphocyte ratio; ULN, upper limit of normal; IrLI, immune-related liver injury.

## Data Availability

The data are not publicly available due to ethical reasons. Further inquiries should be directed to the corresponding authors.

## Supplementary Materials

The following supporting information can be downloaded at: https://www.mdpi.com/article/10.3390/cancers17193157/s1. Figure S1: Flowchart of patient enrollment; Figure S2: Median overall survival (A) and progression-free survival (B) for the entire cohort; Figure S3: Median overall survival (A) and progression-free survival (B) between steroid-treated and non-steroid-treated patients with immune-related liver injury; Figure S4: Median overall survival (A) and progression-free survival (B) between steroid-treated and non-steroid-treated patients with grade 1 immune-related liver injury. The median overall survival (C) and progression-free survival (D) between steroid-treated and non-steroid-treated patients with grade ≥ 2 immune-related liver injury. Figure S5: Stratification analysis of patients receiving locoregional therapy. The median overall survival (A) and progression-free survival (B) between grade 2 immune-related liver injury and other conditions in patients who received locoregional therapy. The median overall survival (C) and progression-free survival (D) between grade 2 immune-related liver injury and other conditions in patients who did not receive locoregional therapy. Figure S6: Stratification analysis of patients receiving antiviral prophylaxis. The median overall survival (A) and progression-free survival (B) between grade 2 immune-related liver injury and other conditions in patients who received antiviral prophylaxis. Median overall survival (C) and progression-free survival (D) between grade 2 immune-related liver injury and other condition in patients who did not receive antiviral prophylaxis; Figure S7: Median overall survival (A) and progression-free survival (B) among patients with different grades of immune-related liver injury after propensity score matching; Table S1: Response of overall enrolled patients evaluated by mRECIST; Table S2: Response according to classification of IrLI’s grade evaluated by mRECIST; Table S3: Factors associated with grade 2 irLI after ICI therapy among patients with irLI; Table S4: Association of other treatment-related adverse events with IrLI; Table S5: Baseline characteristics of patients among different irLI grades after propensity score matching; Table S6: Response according to classification of IrLI’s grade evaluated by mRECIST after propensity score matching; Table S7: Predictors of overall survival after propensity score matching; Table S8: Predictors of progression-free survival after propensity score matching.

## Abbreviations

The following abbreviations are used in this manuscript:

ALT	alanine aminotransferase
AST	aspartate aminotransferase
BCLC	Barcelona Clinic Liver Cancer
CR	complete response; DCR, disease control rate
EHS	extrahepatic spread
HCC	Hepatocellular carcinoma
ICIs	immune checkpoint inhibitors
irLI	immune-related liver injury
LRT	locoregional therapy
ORR	objective response rate
OS	overall survival
PFS	progression-free survival
PVT	portal vein thrombosis
RFA	radiofrequency ablation
SD	stable disease
TACE	transarterial chemoembolization
ULN	upper limit of normal
