# Supplementary material for: Moderate Immune-Related Liver Injury Is a Good Factor in Patients with Hepatoma Under Atezolizumab Plus Bevacizumab
Source: Cancers (Basel). 2025 Sep 28;17(19):3157. doi: 10.3390/cancers17193157 (PMC12523717; doi:10.3390/cancers17193157)
Supplement: Supplementary file 1 [file cancers-17-03157-s001.zip › cancers-3866331-supplementary.pdf]

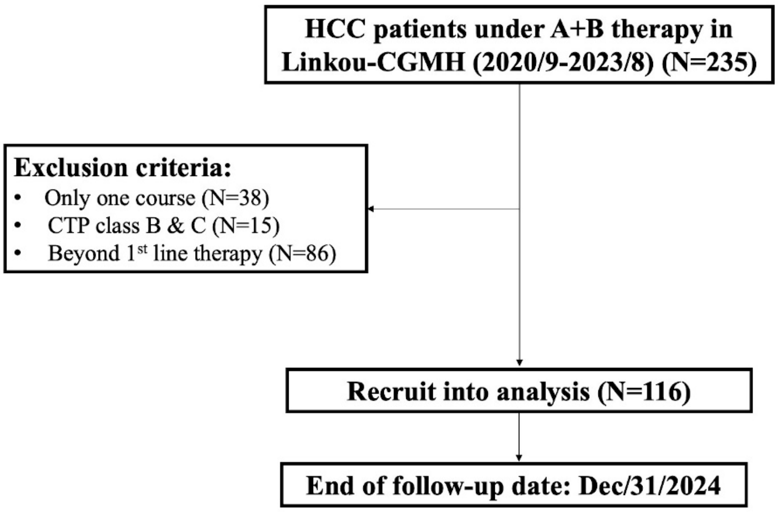

Figure S1. Flowchart of patient enrollment.

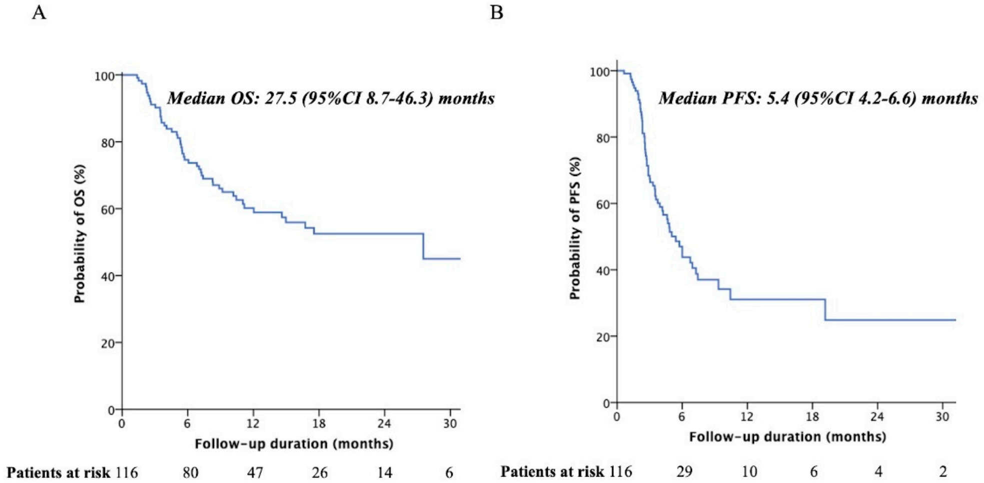

Figure S2. Median overall survival (**A**) and progression-free survival (**B**) for the entire cohort.

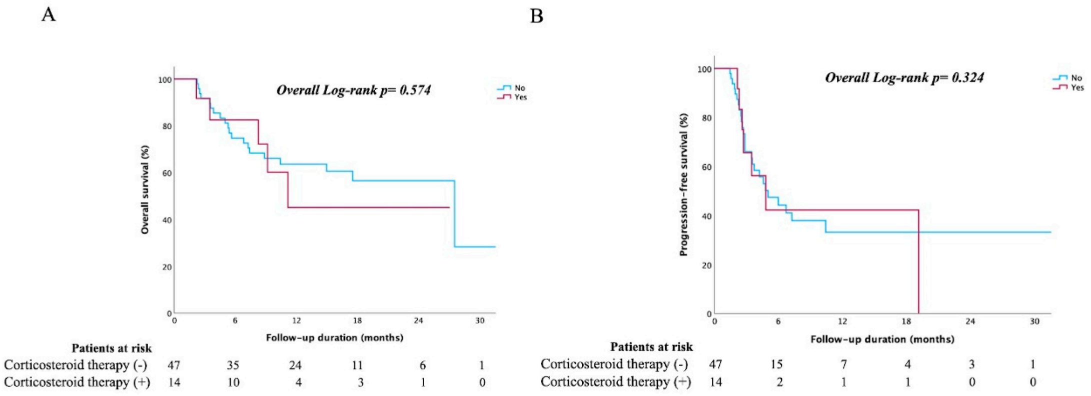

Figure S3. Median overall survival (**A**) and progression-free survival (**B**) between steroid-treated (n = 14) and non-steroid-treated patients (n = 47) with immune-related liver injury.

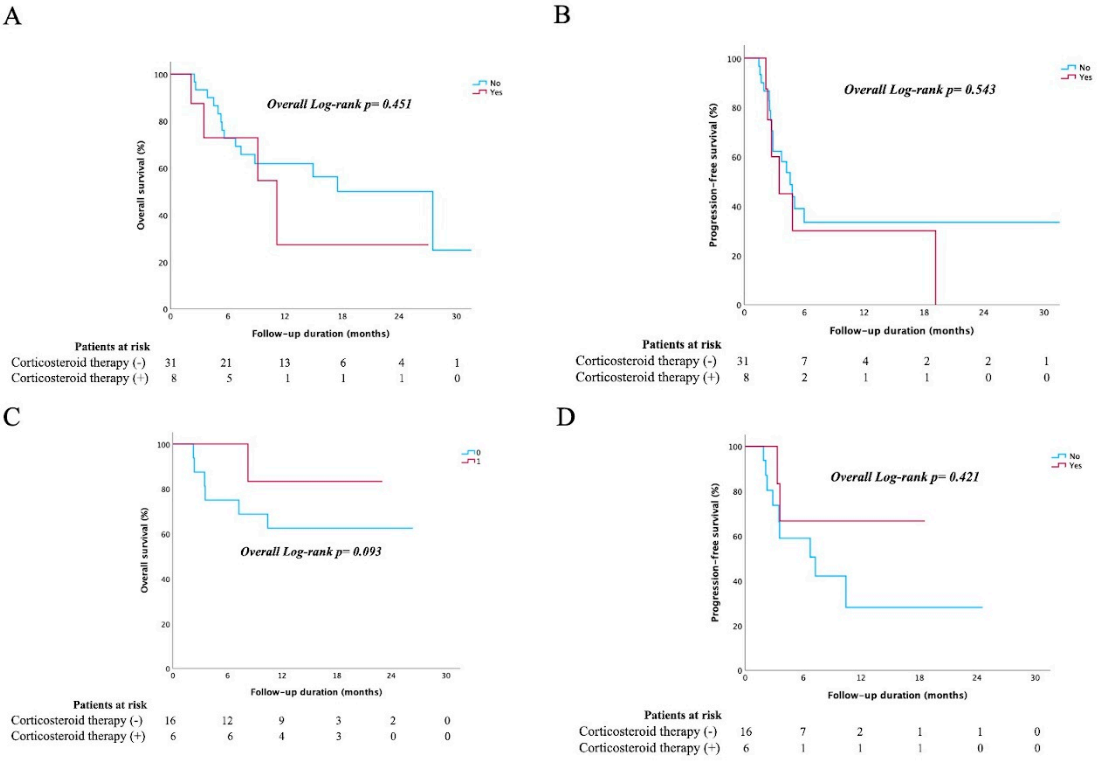

Figure S4. Median overall survival (**A**) and progression-free survival (**B**) between steroid-treated (n = 8) and non-steroid-treated patients (n = 31) with grade 1 immune-related liver injury. The median overall survival (**C**) and progression-free survival (**D**) between steroid-treated (n = 6) and non-steroid-treated patients (n = 16) with grade ≥2 immune-related liver injury.

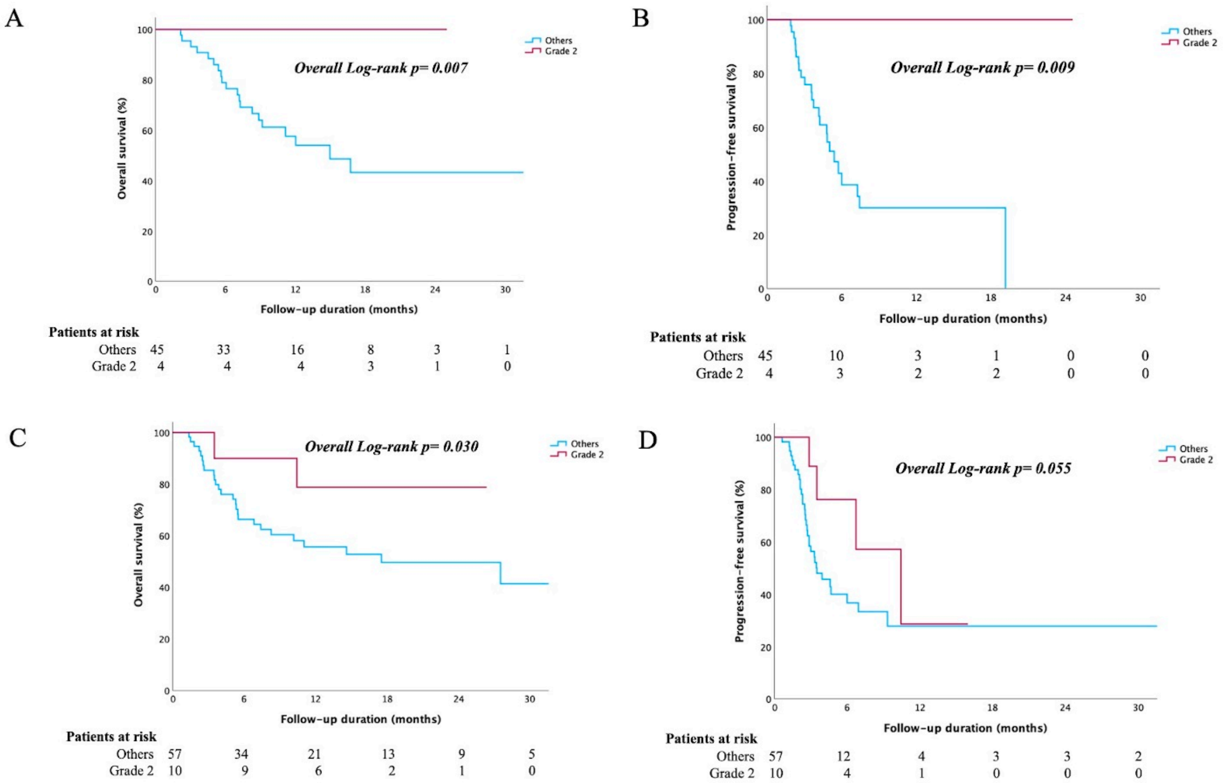

Figure S5. Stratification analysis of patients receiving locoregional therapy. The median overall survival (**A**) and progression-free survival (**B**) between grade 2 immune-related liver injury ($n = 4$) and other conditions ($n = 45$) in patients who received locoregional therapy. The median overall survival (**C**) and progression-free survival (**D**) between grade 2 immune-related liver injury ($n = 10$) and other conditions ($n = 57$) in patients who did not receive locoregional therapy.

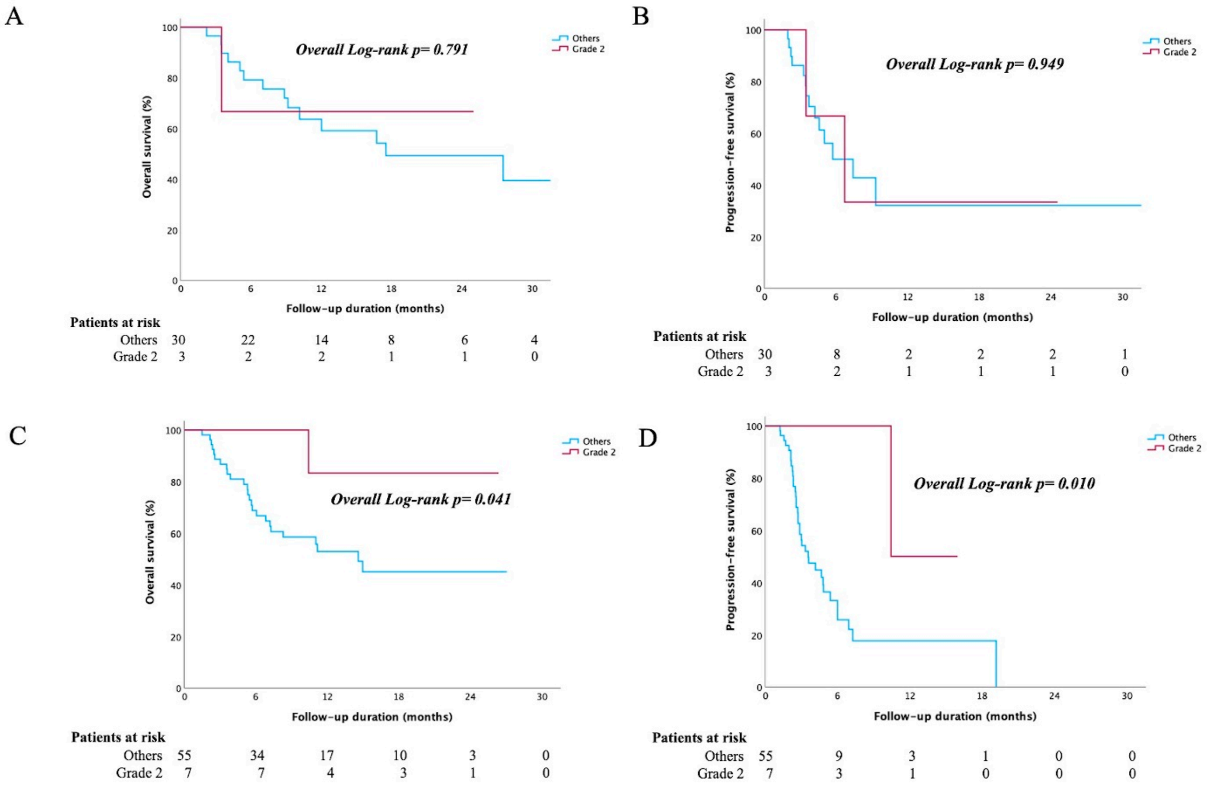

Figure S6. Stratification analysis of patients receiving antiviral prophylaxis. The median overall survival (**A**) and progression-free survival (**B**) between grade 2 immune-related liver injury (*n* = 3) and other conditions (*n* = 30) in patients who received antiviral prophylaxis. Median overall survival (**C**) and progression-free survival (**D**) between grade 2 immune-related liver injury (*n* = 7) and other condition (*n* = 55) in patients who did not receive antiviral prophylaxis.

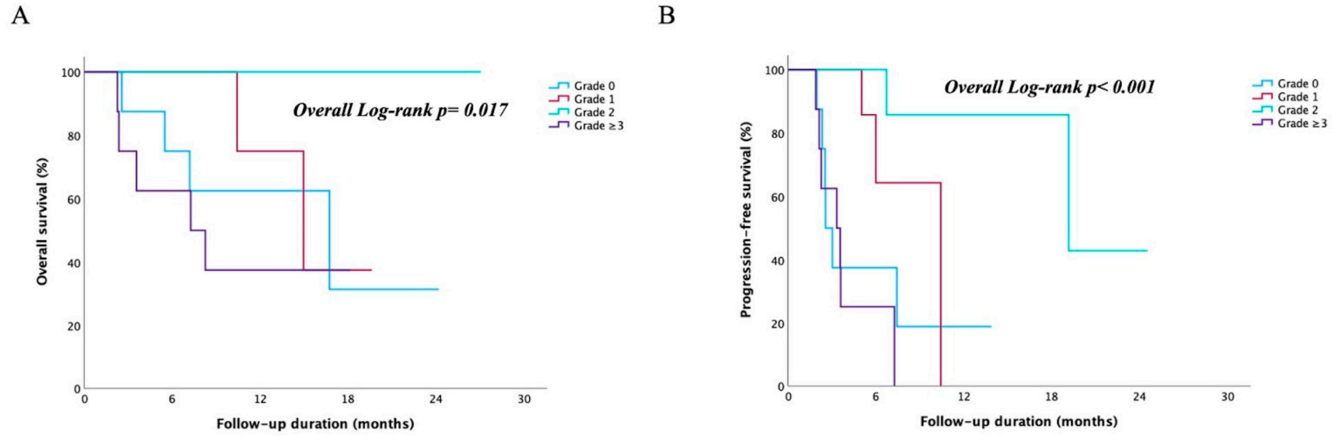

Patients at risk (A)

Grade 0	8	6	3	1	1	0
Grade 1	8	8	3	1	0	0
Grade 2	8	8	7	5	3	0
Grade ≥3	8	5	3	1	0	0

Patients at risk (B)

Grade 0	8	2	1	0	0	0
Grade 1	8	4	0	0	0	0
Grade 2	8	7	5	3	1	0
Grade ≥3	8	1	0	0	0	0

Figure S7. Median overall survival (**A**) and progression-free survival (**B**) among patients with different grades of immune-related liver injury (*n* = 8 in each group) after propensity score matching;.

Table S1. Response of overall enrolled patients evaluated by mRECIST.

Outcome, *n* (%)	Initial response (*n* = 116)	Best response (*n* = 116)
Objective response	41 (35.4)	46 (39.7)
Complete response	3 (2.6)	9 (7.8)
Partial response	38 (32.8)	37 (31.9)
Stable disease	40 (34.5)	36 (31.0)
Disease control rate	81 (69.9)	82 (70.7)
Progressive disease	35 (30.1)	34 (29.3)

Table S2. Response according to classification of IrLI's grade evaluated by mRECIST.

Outcome, *n* (%)	Initial response					Best response				
	Grade 0 (*n* = 55)	Grade 1 (*n* = 39)	Grade 2 (*n* = 14)	Grade ≥ 3 (*n* = 8)	*p*-value	Grade 0 (*n* = 55)	Grade 1 (*n* = 39)	Grade 2 (*n* = 14)	Grade ≥ 3 (*n* = 8)	*p*-value
Objective response	20 (36.4)	13 (33.4)	6 (42.8)	2 (25.0)	0.646	23 (41.8)	14 (35.9)	7 (50.0)	2 (25.0)	0.665
Complete response	1 (1.8)	1 (2.6)	1 (7.1)	0 (0)		6 (10.9)	2 (5.1)	1 (7.1)	0 (0)	
Partial response	19 (34.5)	12 (30.8)	5 (35.7)	2 (25.0)		17 (30.9)	12 (30.8)	6 (42.9)	2 (25.0	
Stable disease	17 (30.9)	14 (35.9)	7 (50.0)	2 (25.0)		14 (25.5)	14 (35.9)	6 (42.9)	2 (25.0)	

Disease control rate	37 (67.3)	27 (69.2)	13 (92.9)	4 (50.0)	0.079	37 (67.3)	28 (71.8)	13 (92.9)	4 (50.0)	0.053
Progressive disease	18 (32.7)	12 (30.8)	1 (7.1)	4 (50.0)		18 (32.7)	11 (28.2)	1 (7.1)	4 (50.0)	

Table S3. Factors associated with grade 2 irLI after ICI therapy among patients with irLI.

Variables	Univariate			Multivariate		
	HR	95%CI	*p* value	HR	95%CI	*p* value
Age ≥ 65 y/o (vs. < 65 y/o)	0.651	0.389-1.092	0.104			
Male (vs. female)	1.382	0.651-2.935	0.399			
Viral infection (vs. others)	1.700	0.879-3.287	0.115			
Antiviral therapy (vs. no)	0.598	0.309-0.957	0.047	0.726	0.364-0.948	0.043
ALBI grade II (vs. I)	1.253	0.752-2.090	0.387			
Baseline AST or ALT > ULN (vs. No)	1.701	1.115-3.551	0.047	2.703	0.903-8.093	0.075
Tumor size	0.949	0.834-1.079	0.424			
Tumor numbers ≥ 3 (vs. < 3)	0.663	0.219-2.002	0.466			
Portal vein thrombosis (vs. No)	1.325	0.792-2.215	0.283			
Extrahepatic metastasis (vs. No)	1.245	0.738-2.099	0.412			
AFP ≥ 400 ng/ml (vs.< 400 ng/ml)	1.018	0.607-1.708	0.946			
BCLC stage B (vs. C)	0.886	0.484-1.621	0.694			
Prior LRT (vs. No)	0.766	0.453-1.294	0.319			
Combination with LRT (vs. No)	1.590	1.028-2.724	0.042	1.230	1.070-2.257	0.045
ICI treatment duration	1.005	0.895-1.128	0.930			

Abbreviations: AFP, Alpha-fetoprotein; ALT, alanine aminotransferase; AST, aspartate aminotransferase; BCLC, Barcelona Clinic Liver Cancer classification; LRT, locoregional therapy; NLR, Neutrophil-to-lymphocyte ratio; ULN, upper limit of normal; IrLI, immune-related liver injury.

Table S4. Association of other treatment-related adverse events with IrLI.

Adverse events	Overall (*n* = 116)		Grade 0 (*n* = 55)		Grade 1 (*n* = 39)		Grade 2 (*n* = 14)		Grade ≥ 3 (*n* = 8)	
	Any grade	Grade ≥ 3	Any grade	Grade ≥ 3	Any grade	Grade ≥ 3	Any grade	Grade ≥ 3	Any grade	Grade ≥ 3
Any adverse events	80 (69.0)	18 (15.5)	19 (34.5)	4 (7.2)	39 (100)	3 (7.7)	14 (100)	3 (21.4)	8 (100)	3 (37.5)
Hypertension	21 (18.1)	5 (4.3)	12 (21.8)	4 (7.3)	6 (15.4)	1 (2.6)	2 (14.3)	0 (0)	1 (12.5)	0 (0)
Proteinuria	28 (24.1)	4 (3.4)	4 (7.3)	0 (0)	15 (38.5)	2 (5.1)	6 (42.9)	2 (14.3)	3 (37.5)	0 (0)
Bilirubin elevation	33 (28.4)	6 (5.2)	7 (12.7)	0 (0)	18 (46.2)	2 (5.1)	4 (28.6)	1 (7.1)	4 (50.0)	3 (37.5)
Thrombocytopenia	33 (28.4)	2 (1.7)	14 (25.5)	0 (0)	12 (30.8)	0 (0)	5 (35.7)	2 (14.3)	2 (25.0)	0 (0)
GI bleeding	10 (8.6)	4 (3.4)	5 (9.1)	3 (5.5)	4 (10.3)	0 (0)	0 (0)	0 (0)	1 (12.5)	1 (12.5)
Skin rash	2 (1.7)	0 (0)	1 (1.8)	0 (0)	1 (2.6)	0 (0)	0 (0)	0 (0)	0 (0)	0 (0)
Thyroid disorder	23 (19.8)	0 (0)	8 (14.5)	0 (0)	12 (30.8)	0 (0)	1 (7.1)	0 (0)	2 (25.0)	0

Table S5. Baseline characteristics of patients among different irLI grades after propensity score matching.

Variables	IrLI grade 0 (*n* = 8)	IrLI grade 1 (*n* = 8)	IrLI grade 2 (*n* = 8)	IrLI grade ≥3 (*n* = 8)	*p* value
Age (year-old)	68.2 (IQR 59.0-83.2)	62.7 (IQR 38.9-75.3)	70.3 (IQR 63.0-73.2)	68.4 (IQR 62.2-70.5)	0.731
Male gender, *n* (%)	6 (75.0)	6 (75.0)	7 (87.5)	8 (100)	0.556

HBV/HCV/NBNC, *n* (%)	6/1/1 (75.0/12.5/12.5)	6/1/1 (75.0/12.5/12.5)	5/1/2 (62.5/12.5/25.0)	5/1/2 (62.5/12.5/25.0)	0.991
NLR	3.12 (IQR 2.61-4.51)	4.60 (IQR 3.84-7.18)	3.02 (IQR 2.30-5.54)	3.92 (IQR 2.49-7.89)	0.612
AST (IU/ml)	72 (IQR 37-124)	41 (IQR 30-122)	50 (IQR 37-68)	60 (IQR 45-110)	0.623
ALT (IU/ml)	55 (IQR 40-76)	41 (IQR 20-67)	37 (IQR 22-72)	56 (IQR 34-105)	0.631
Baseline AST or ALT > ULN, *n* (%)	7 (87.5)	7 (87.5)	7 (87.5)	7 (87.5)	-
Bilirubin total (mg/dL)	0.85 (IQR 0.73-1.43)	0.74 (IQR 0.43-1.43)	0.65 (IQR 0.50-1.15)	0.85 (IQR 0.53-1.50)	0.559
Albumin (g/dL)	3.68 (IQR 3.30-4.06)	3.57 (IQR 3.13-3.88)	3.71 (IQR 3.32-4.06)	3.55 (IQR 2.94-3.96)	0.647
Platelet (10^3/μL)	248 (IQR 174-286)	306 (IQR 154-413)	233 (IQR 160-272)	262 (IQR 147-361)	0.517
ALBI grade I/II, *n* (%)	2/6 (25.0/75.0)	2/6 (25.0/75.0)	2/6 (25.0/75.0)	2/6 (25.0/75.0)	-
Portal vein thrombosis, *n* (%)	4 (50.0)	5 (62.5)	4 (50.0)	4 (50.0)	0.797
Esophageal varices, *n* (%)	0 (0)	1 (12.5)	2 (25.0)	2 (25.0)	0.456
Extrahepatic metastasis, *n* (%)	2 (25.0)	3 (37.5)	2 (25.0)	2 (25.0)	0.821
BCLC stage B/C, *n* (%)	2/6 (75.0/25.0)	2/6 (75.0/25.0)	2/6 (75.0/25.0)	2/6 (75.0/25.0)	-
Target tumor size, cm	9.6 (IQR 2.8-10.5)	10.0 (IQR 7.6-16.8)	8.5 (IQR 3.2-13.2)	8.9 (IQR 7.5-9.9)	0.601
Tumor number ≥3, *n* (%)	4 (50.0)	5 (62.5)	5 (62.5)	4 (50.0)	0.706
AFP (ng/ml)	75 (IQR 10-4967)	72 (IQR 6-46202)	65 (IQR 13-425)	70 (IQR 15-3108)	0.764
Combination with LRT, *n* (%)	4 (50.0)	4 (50.0)	4 (50.0)	4 (50.0)	-
Prior LRT, *n* (%)	5 (62.5)	3 (37.5)	4 (50.0)	5 (62.5)	0.710
ICI treatment duration (months)	2.6 (IQR 1.4-6.2)	2.8 (IQR 1.4-4.7)	2.3 (IQR 1.8-6.2)	3.2 (IQR 1.2-4.8)	0.891

Abbreviations: AFP, Alpha-fetoprotein; ALT, alanine aminotransferase; AST, aspartate aminotransferase; BCLC, Barcelona Clinic Liver Cancer classification; HBV, hepatitis B virus; irLI, immune-related liver injury; LRT, locoregional therapy; NLR, Neutrophil-to-lymphocyte ratio; ULN, upper limit of normal.

Table S6. Response according to classification of IrLI's grade evaluated by mRECIST after propensity score matching.

SOutcome, *n* (%)	Initial response					Best response				
	Grade 0 (*n* = 8)	Grade 1 (*n* = 8)	Grade 2 (*n* = 8)	Grade ≥3 (*n* = 8)	*p*-value	Grade 0 (*n* = 8)	Grade 1 (*n* = 8)	Grade 2 (*n* = 8)	Grade ≥3 (*n* = 8)	*p*-value
Objective response	3 (37.5)	4 (50.0)	4 (50.0)	2 (25.0)	0.482	3 (37.5)	4 (50.0)	5 (62.5)	2 (25.0)	0.058
Complete response	0 (0)	0 (0)	0 (0)	0 (0)		1 (12.5)	1 (12.5)	1 (12.5)	0 (0)	
Partial response	3 (37.5)	4 (50.0)	4 (50.0)	2 (25.0)		2 (25.0)	3 (37.5)	4 (50.0)	2 (25.0)	
Stable disease	0 (0)	4 (50.0)	4 (50.0)	2 (25.0)		0 (0)	4 (50.0)	3 (37.5)	2 (25.0)	
Disease control rate	3 (37.5)	8 (100)	8 (100)	4 (50.0)	0.024	3 (37.5)	8 (100)	8 (100)	4 (50.0)	0.005
Progressive disease	5 (62.5)	0 (0)	0 (0)	4 (50.0)		5 (62.5)	0 (0)	0 (0)	4 (50.0)	

Table S7. Predictors of overall survival after propensity score matching.

Variables	Univariate			Multivariate		
	HR	95%CI	*p* value	HR	95%CI	*p* value
Age ≥ 65 years old (vs. < 65 years old)	0.393	0.104-1.485	0.169			
Male (vs. female)	0.599	0.126-2.837	0.518			
Viral infection (vs. others)	1.084	0.234-5.024	0.918			
Antiviral therapy (vs. no)	0.223	0.028-1.791	0.158			

ALBI grade II (vs. I)	1.434	0.409-5.032	0.573			
Baseline AST or ALT > ULN (vs. No)	27.17	0.027-27185	0.349			
BCLC stage C (vs. stage A/B)	2.040	0.436-9.537	0.365			
Tumor size	1.003	0.895-1.124	0.964			
Tumor numbers ≥ 3 (vs. < 3)	1.686	0.514-5.527	0.389			
Portal vein thrombosis (vs. No)	1.516	0.441-5.204	0.509			
Extrahepatic metastasis (vs. No)	1.001	0.265-3.786	0.999			
AFP ≥ 400 ng/ml (vs.< 400 ng/ml)	1.411	0.430-4.633	0.570			
Prior LRT (vs. No)	0.771	0.234-2.549	0.670			
Combination with LRT (vs. No)	0.719	0.217-2.381	0.589			
Grade 2 irLI (vs. others)	0.221	0.028-0.933	0.041			
Corticosteroid therapy (vs. No)	0.294	0.038-2.301	0.243			
ICI treatment duration	0.805	0.653-1.294	0.084			

Abbreviations: AFP, Alpha-fetoprotein; ALT, alanine aminotransferase; AST, aspartate aminotransferase; LRT, locoregional therapy; ULN, upper limit of normal; IrLI, immune-related liver injury.

Table S8. Predictors of progression-free survival after propensity score matching.

Variables	Univariate			Multivariate		
	**HR**	**95%CI**	***p* value**	**HR**	**95%CI**	***p* value**
Age ≥ 65 years old (vs. < 65 years old)	0.334	0.118-1.044	0.069			
Male (vs. female)	0.507	0.162-1.581	0.242			
Viral infection (vs. others)	1.821	0.414-8.006	0.427			
Antiviral therapy (vs. no)	0.944	0.325-2.744	0.916			
ALBI grade II (vs. I)	1.919	0.664-5.542	0.228			
Baseline AST or ALT > ULN (vs. No)	3.399	0.449-25.76	0.236			
BCLC stage C (vs. stage A/B)	0.919	0.340-2.482	0.868			
Tumor size	0.947	0.858-1.046	0.283			
Tumor numbers ≥ 3 (vs. < 3)	2.511	0.818-7.709	0.108			
Portal vein thrombosis (vs. No)	1.457	0.574-3.698	0.428			
Extrahepatic metastasis (vs. No)	1.090	0.354-3.356	0.881			
AFP ≥ 400 ng/ml (vs.< 400 ng/ml)	1.239	0.467-3.286	0.667			
Prior LRT (vs. No)	0.993	0.394-2.506	0.989			
Combination with LRT (vs. No)	0.749	0.295-1.901	0.544			
Grade 2 irLI (vs. others)	0.208	0.047-0.915	0.038			
Corticosteroid therapy (vs. No)	0.609	0.173-2.140	0.439			
ICI treatment duration	0.709	0.576-1.174	0.081			

Abbreviations: AFP, Alpha-fetoprotein; ALT, alanine aminotransferase; AST, aspartate aminotransferase; NLR, Neutrophil-to-lymphocyte ratio; ULN, upper limit of normal; IrLI, immune-related liver injury.
